# Encapsulated Omental Necrosis as an Unexpected Postoperative Finding: A Case Report

**DOI:** 10.3390/medicina57090865

**Published:** 2021-08-24

**Authors:** Milica Mitrovic, Dejan Velickovic, Marjan Micev, Vladimir Sljukic, Petar Djuric, Boris Tadic, Ognjan Skrobic, Jelena Djokic Kovac

**Affiliations:** 1 Center for Radiology and Magnetic Resonance Imaging, Clinical Centre of Serbia, Pasterova No. 2, 11000 Belgrade, Serbia; dr_milica@yahoo.com (M.M.); jelenadjokickovac@gmail.com (J.D.K.); 2 Department of Stomach and Esophageal Surgery, Clinic for Digestive Surgery, Clinical Centre of Serbia, Koste Todorovica Street No. 6, 11000 Belgrade, Serbia; velickovicdejan@gmail.com (D.V.); vlada.sljukic@gmail.com (V.S.); skrobico@gmail.com (O.S.); 3 Department for Surgery, Faculty of Medicine, University of Belgrade, Dr Subotica No. 8, 11000 Belgrade, Serbia; 4 Department for Pathology, Clinic for Digestive Surgery, Clinical Centre of Serbia, Dr Subotica No. 8, 11000 Belgrade, Serbia; micevm@gmail.com; 5 Center for Nephrology, University Hospital Zvezdara, Dimitrija Tucovica No. 161, 11000 Belgrade, Serbia; djuricmed@gmail.com; 6 Department for HBP Surgery, Clinic for Digestive Surgery, Clinical Centre of Serbia, Koste Todorovica Street No. 6, 11000 Belgrade, Serbia; 7 Department for Radiology, Faculty of Medicine, University of Belgrade, Dr Subotica No. 8, 11000 Belgrade, Serbia

**Keywords:** appendagitis, omental infarction, steatonecrosis, gastric surgery, fat necrosis

## Abstract

Postsurgical fat necrosis is a frequent finding in abdominal cross-sectional imaging. Epiploic appendagitis and omental infarction are a result of torsion or vascular occlusion. Surgery or pancreatitis are conditions that can have a traumatic and ischemic effect on fatty tissue. The imaging appearances may raise concerns for recurrent malignancy, but percutaneous biopsy and diagnostic follow-up assist in the accurate diagnosis of omental infarction. Herein we describe a case of encapsulated omental necrosis temporally related to gastric surgery. Preoperative CT and MRI findings showed the characteristics of encapsulated, postcontrast nonviable tumefaction in the epigastrium without clear imaging features of malignancy. Due to the size of the lesion and the patient’s primary disease, tumor recurrence could not be completely ruled out, and the patient underwent surgery. Histopathological analysis confirmed the diagnosis of steatonecrosis of the omentum.

## 1. Introduction

Intra-abdominal fat is metabolically active tissue that may undergo necrosis due to the torsion of an epiploic appendage, infarction of the greater omentum, and as a consequence of trauma or pancreatitis [1]. Epiploic appendagitis and omental infarction are conditions that manifest with abdominal pain and mimic findings of acute abdomen. On the other hand, encapsulated fat necrosis occurs due to traumatic, ischemic insult that causes fat degeneration with a specific imaging appearance that may be easily misdiagnosed with malignancy [2].

## 2. Case Report

**Figure 1:**
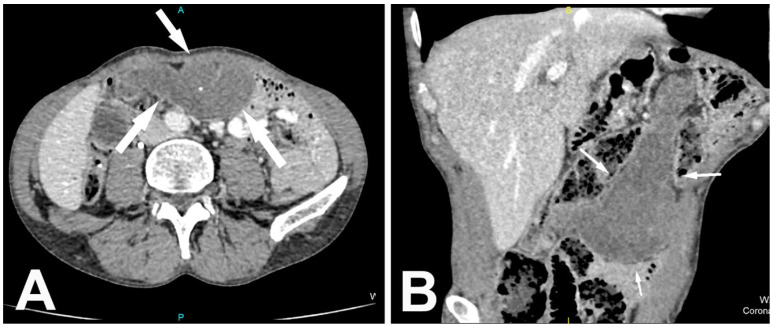
MDCT axial (**A**) and coronal (**B**) views show in the region of the previous operation an encapsulated, triangular mass without postcontrast opacification, involving practically the entire omentum. Axial scan reveals punctiform microcalcification and discrete internal septations.

A 56-year-old male patient was admitted to our hospital with mild abdominal pain and palpable mass in the epigastric region. One year before, the patient underwent subtotal esophagectomy with transmediastinal gastroplasty and cervical esophago–gastro anastomosis due to squamous cell carcinoma of the proximal segment of the thoracic esophagus. Three cycles of chemotherapy were performed during which abdominal pain persisted. When the patient noted the palpable lump in the upper abdomen, he consulted a surgeon. Ultrasound examination of the abdomen showed a sharply delineated, incompressible, heteroechogenic tumefaction in the epigastric region. No inflammatory changes in the surrounding adipose tissue or the presence of free fluid were visualized. There were no signs of internal vascularization. Contrast-enhanced computed tomography (CT) was performed, and a hypodense, well-defined, clearly demarcated omental mass was revealed (Figure 1A,B).

**Figure 2:**
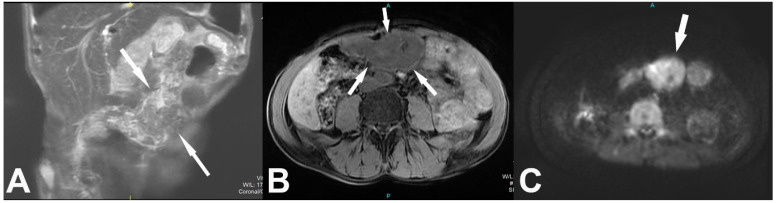
MRI images: T2W coronal (**A**), T1W axial view (**B**), and DWI (**C**) demonstrate clearly delineated mass with thin fibrous capsula in the epigastric region and irregular internal zones of higher signal intensity at T2W, predominantly lower IS at T1W and restricted diffusion. DWI—diffusion-weighted imaging; IS—lower signal intensity.

There was no significant postcontrast enhancement of the lesion, which showed discrete interspersed fat attenuation foci and several punctiform microcalcifications. There were no signs of the infiltrative character of the lesion or signs of angioinvasion or locoregional lymphadenopathy. Furthermore, an abdominal MRI exam showed a (T2-weighted) T2W heterointensive mass with irregular areas of higher signal intensity (Figure 2A). The lesion was mainly (T1-weighted) T1W hypointense, surrounded by a hyperintense capsule and discrete fat content (Figure 2B). The lesion displayed restricted diffusion, which was observed as high signal intensity on diffusion-weighted imaging (DWI) with corresponding reduced apparent diffusion coefficient (ADC) values (Figure 2C).

**Figure 3:**
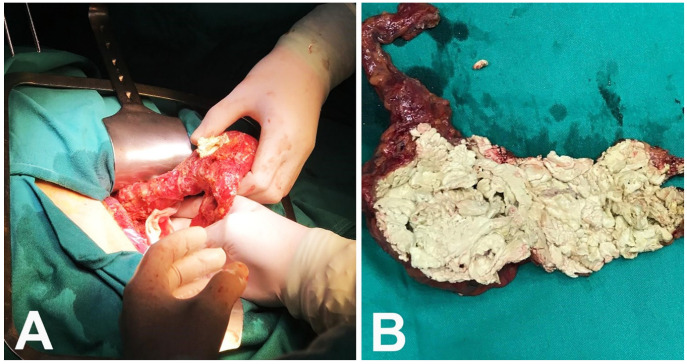
Intraoperative sight: completely excided epigastric mass (**A**) and resected operative specimen (**B**).

Imaging features were predominantly benign, but due to the patient’s primary disease and lesion diameter, the malignancy could not be ruled out with certainty. The patient underwent open surgery, and the tumor-like mass was completely excided (Figure 3A,B).

**Figure 4:**
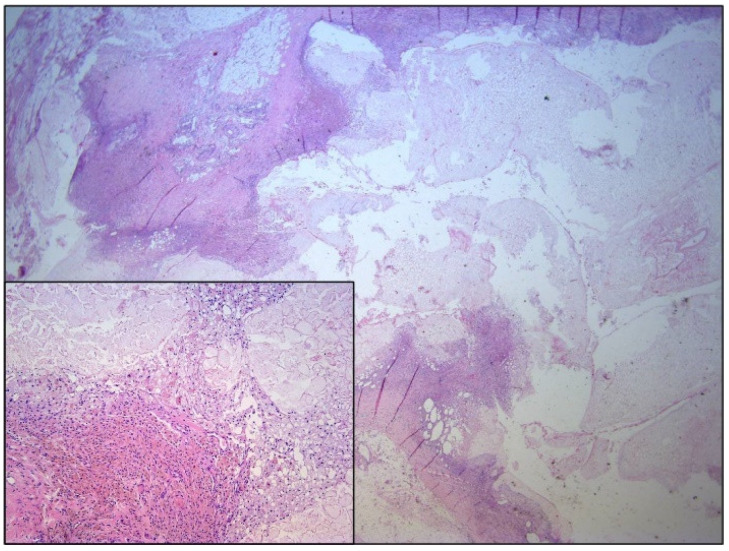
Nodular cystic fat necrosis histologically shows prominent central liponecrosis and irregular fibro-inflammatory peripheral zone, which on higher magnification reveals mostly foamy macrophages, giant cells, and fibroblasts (inlet).

The histopathological finding correlated with encapsulated omental necrosis in the form of a secondary omental infarction that probably occurred as a consequence of an interruption to omental blood supply due to surgical manipulation at the time of gastric mobilization (Figure 4). The postoperative course was uneventful. He was discharged from hospital 7 days after surgery.

## 3. Discussion

The greater omentum is a two-layered fold of the peritoneum arising from the greater curvature of the stomach without any attachment on three sides. It is highly mobile and susceptible to disruption of the omental blood supply, which can occur due to surgical manipulation during gastric or colonic mobilization, or by subsequent compromise during postoperative healing [3,4].

Fat necrosis occurs as a result of infarction of greater omentum, torsion of an epiploic appendage, or due to trauma or pancreatitis. Primary omental infarction can also occur in a hypercoagulable state, congestive heart failure, or thrombosis of the superior mesenteric vein or artery in obesity and professional marathon runners. It most often occurs in the right hemiabdomen due to the higher frequency of torsion of the right omentum [5].

The most common symptom of omental infarction and acute appendagitis is abdominal pain that may be the cause of acute abdomen. The widespread use of CT scans in the evaluation of emergencies has increased the diagnostic confirmation of these conditions.

Clinical presentation can also be completely asymptomatic, so these disorders are quite accidentally detected on surveillance imaging [1,6].

Accurate diagnosis is very important because most of these patients are treated conservatively. Secondary omental infarction is a consequence of surgery and can easily be misinterpreted in further follow-up as peritoneal dissemination of the disease or recurrence of malignancy [3].

The ultrasound examination in primary omental infarction indicates a hyperechogenic mass with a very discrete hypoechogenic rim and edematous surrounding adipose tissue corresponding to inflammation. Reactive lymph nodes can often be observed. The mass shows no sign of internal vascularization or compressibility [7].

CT presentation of primary omental infarction is an irregularly demarcated zone of soft-tissue stranding, predominantly fat density, and sometimes over 5 cm in size. Imaging appearance of the secondary omental infarction has been classified into four subtypes. Type 1 demonstrates an ill-defined fat attenuation lesion, type 2 shows a well-defined peripheral rim enhancement, while type 3 contains a partial fat component in a predominantly heterogeneous structure of the lesion, and type 4 represents a clearly delineated heterodense mass without a fat component [8].

Encapsulated fat has a thin fibrous capsule and can be further complicated by inflammation and abscess formation. Calcifications are often detected intralesionally. It can easily be misdiagnosed with a malignant lesion because the capsule can show postcontrast viability on CT imaging. In such cases, CT shows an encapsulated mass, usually in the omentum. A fat component is not always observed. A discrete postcontrast capsule opacification can be observed as well as irregular internal septations [1]. There is no infiltration of surrounding structures, and the lesion does not increase in size during the time. It is temporally related to surgery and has a tendency to decrease in size on surveillance imaging. Nevertheless, the main differential diagnosis is well-differentiated liposarcoma [2,9].

On MRI, encapsulated fat necrosis is observed as a sharply demarcated lesion heterogeneous on T2-weighted images without complete fat suppression and mostly low IS on T1-weighted images. It can demonstrate high signal intensity on DWI and, therefore, resemble malignant lesions. It may present a variable degree of FDG uptake on PET/CT [1,10].

One of the conditions in which adipose tissue necrosis can also be observed is pancreatitis, in which the process of fatty saponification may occur. Lipohypertrophy within the subcutaneous tissue has similar imaging characteristics and is found in patients who inject insulin injections [11,12].

To our knowledge, there are no cases of such extensive encapsulated omental necrosis described in the previous literature. The size of the lesion and its bizarre imaging features have raised suspicion of disease recurrence. Therefore, it is very important to keep in mind the complete clinical history of the patient as well as previous imaging studies in order to avoid further unnecessary examinations or even inappropriate treatments [3].

## 4. Conclusions

The diagnostic features of encapsulated fat necrosis can be quite various and atypical. They can raise the suspicion of tumor recurrence or secondary dissemination of malignancy. Percutaneous biopsy, correlation of findings with previous imaging, and follow-up can help to avoid misinterpretation of such lesions and unnecessary procedures. In most cases, spontaneous resolution is expected unless it is complicated by inflammation that requires additional radiological and possibly surgical treatment.

## Data Availability

All the data are available from the corresponding author upon reasonable request.
